# N^1^-Methyladenosine modification of mRNA regulates neuronal gene expression and oxygen glucose deprivation/reoxygenation induction

**DOI:** 10.1038/s41420-023-01458-2

**Published:** 2023-05-12

**Authors:** Zhangyang Qi, Chi Zhang, Huan Jian, Mengfan Hou, Yongfu Lou, Yi Kang, Wei Wang, Yigang Lv, Shenghui Shang, Chaoyu Wang, Xueying Li, Shiqing Feng, Hengxing Zhou

**Affiliations:** 1 Department of Orthopaedics, Qilu Hospital, Shandong University Centre for Orthopaedics, Advanced Medical Research Institute, Cheeloo College of Medicine, Shandong University, Jinan, Shandong 250012 P.R. China; 2 Department of Orthopaedics, Tianjin Medical University General Hospital, International Science and Technology Cooperation Base of Spinal Cord Injury, Tianjin Key Laboratory of Spine and Spinal Cord, Tianjin, 300052 P.R. China; 3 Shandong University Centre for Orthopaedics, Advanced Medical Research Institute, Cheeloo College of Medicine, Shandong University, Jinan, Shandong 250012 P.R. China; 4 Center for Reproductive Medicine, Shandong University, Jinan, Shandong 250012 China

**Keywords:** Cellular neuroscience, Epigenomics

## Abstract

N^1^-Methyladenosine (m1A) is an abundant modification of transcripts, plays important roles in regulating mRNA structure and translation efficiency, and is dynamically regulated under stress. However, the characteristics and functions of mRNA m1A modification in primary neurons and oxygen glucose deprivation/reoxygenation (OGD/R) induced remain unclear. We first constructed a mouse cortical neuron OGD/R model and then used methylated RNA immunoprecipitation (MeRIP) and sequencing technology to demonstrate that m1A modification is abundant in neuron mRNAs and dynamically regulated during OGD/R induction. Our study suggests that Trmt10c, Alkbh3, and Ythdf3 may be m1A-regulating enzymes in neurons during OGD/R induction. The level and pattern of m1A modification change significantly during OGD/R induction, and differential methylation is closely associated with the nervous system. Our findings show that m1A peaks in cortical neurons aggregate at both the 5’ and 3’ untranslated regions. m1A modification can regulate gene expression, and peaks in different regions have different effects on gene expression. By analysing m1A-seq and RNA-seq data, we show a positive correlation between differentially methylated m1A peaks and gene expression. The correlation was verified by using qRT-PCR and MeRIP-RT-PCR. Moreover, we selected human tissue samples from Parkinson’s disease (PD) and Alzheimer’s disease (AD) patients from the Gene Expression Comprehensive (GEO) database to analyse the selected differentially expressed genes (DEGs) and differential methylation modification regulatory enzymes, respectively, and found similar differential expression results. We highlight the potential relationship between m1A modification and neuronal apoptosis following OGD/R induction. Furthermore, by mapping mouse cortical neurons and OGD/R-induced modification characteristics, we reveal the important role of m1A modification in OGD/R and gene expression regulation, providing new ideas for research on neurological damage.

## Introduction

More than 160 post-transcriptional modifications can occur in eukaryotes, and these modifications play important roles in regulating a number of biological processes, such as embryonic development, cell signal transduction, and axon regeneration [1–4]. Previous studies have shown that N^1^-methyladenosine (m1A) is widely present in tRNA and rRNA and affects the structural stability of RNA by altering its secondary structure, thereby affecting protein translation efficiency [5–8]. Recent studies have shown that m1A is also abundant in the mRNA of eukaryotic cells, enriched in the 5’ untranslated region (UTR) and near the start codon of the mRNA, and can promote protein translation [9]. Under the actions of the writers TRMT6/TRMT61A and the eraser ALKBH3, the level of transcript methylation can be dynamically adjusted [10, 11]. Furthermore, m1A modification of transcripts is also dynamically regulated under H_2_O_2_ stimulation and starvation conditions [9, 11], suggesting that m1A modification participates in regulating oxidative damage. In addition, m1A modification of mRNA is involved in the signal transduction process of tumor invasion [12] and is closely associated with plant growth and development [13], indicating that m1A modification of mRNA affects multiple biological processes [14].

Ischaemia-reperfusion injury (IRI) is an important pathophysiological process of a variety of neurological diseases [15, 16]. The lack of oxygen and glucose and sudden recovery caused by IRI can cause neuronal energy metabolism disorders, reduce ATP production, increase free radical production, and cause neuronal oxidative damage, leading to neuronal apoptosis [17–20]. The oxygen glucose deprivation/reoxygenation (OGD/R) model is an important tool that can be used to study nervous system IRI and has been widely used in a variety of diseases, such as traumatic brain injury (TBI) [21], spinal cord injury (SCI) [22], stroke [23, 24], Alzheimer’s disease (AD) [25], Parkinson’s disease (PD) [26] and other central nervous diseases. However, the features and functions of m1A in neurons and OGD/R injury have not yet been reported.

In the present study, we obtained high-quality primary mouse cortical neurons and constructed a neuronal OGD/R model. Using m1A antibody immunoprecipitation sequencing (MeRIP-seq) technology [9, 11], we performed transcriptome-wide profiling of m1A of mRNA from primary mouse cortical neurons. Our results suggest that m1A modification is abundant in neuron mRNA. During OGD/R induction, the level and pattern mRNA m1A modification was significantly altered, OGD/R-induced methylation modification peaks were enriched in neurological disease-related pathways, and methylation modification levels were closely associated with gene expression. In summary, the results of the present study reveal the dynamic and important role of m1A modification in the mRNA of cortical neurons and OGD/R induction and demonstrate a new mechanism involved in nervous system damage.

## Results and discussion

### Construction of the OGD/R model of primary mouse cortical neurons

**Fig. 1:**
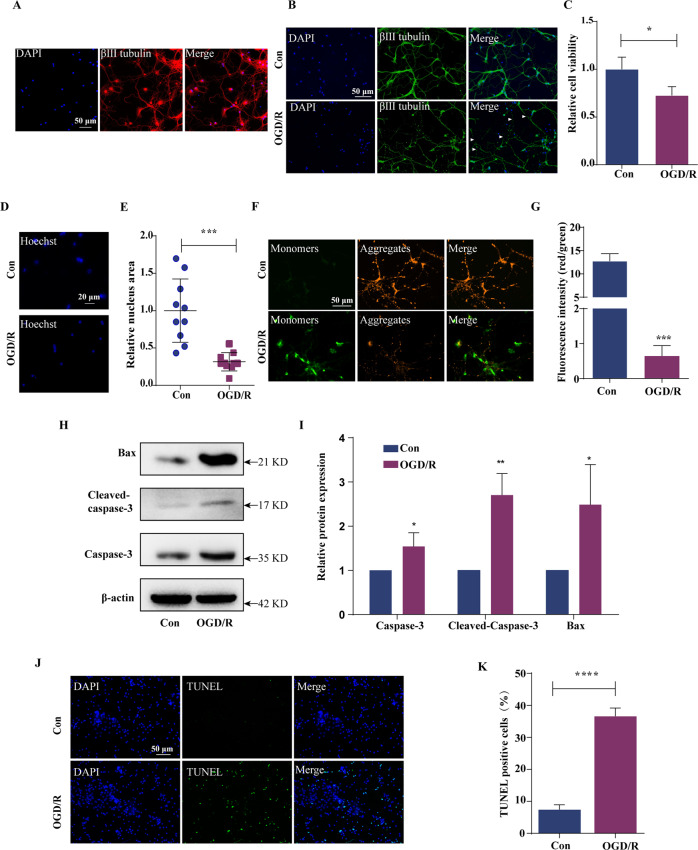
Construction of the OGD/R model of primary mouse cortical neurons. **A** Identification of primary mouse cortical neurons. Blue: DAPI; green: GFAP; red: β-III tubulin. **B** Immunofluorescence of primary neurons induced by OGD/R. **C** CCK-8 assay of the relative viability of neurons induced by OGD/R. **D**, **E** The changes in the nucleus during OGD/R induction were observed by staining with Hoechst. **F**, **G** JC-1 staining image showing changes in mitochondrial membrane potential during OGD/R induction. **H**, **I** Western blot analysis showing the protein expression of caspase-3, Bax, and cleaved Caspase-3 during OGD/R induction. Caspase-3, Bax, and Cleaved caspase-3 expression was quantified and normalised to that of β-actin. **J**, **K** TUNEL staining showing apoptotic cells before and after OGD/R induction. The proportion of TUNEL-positive cells is the percentage of cells with green fluorescence (TUNEL) to those with blue fluorescence (DAPI). Scale bar: 50 µm. Data were analysed using Student’s *t* test. The data are presented as the means ± SD, *n* = 3. *p < 0.05, **p < 0.01, ***p < 0.001.

On the sixth day of cell culture, immunofluorescence was used to determine the purity of the cultured neurons. β-III Tubulin is a neuronal skeleton protein that is used to mark immature neurons [27] (Fig. 1A). Through immunofluorescence analysis, we observed that neurons were induced by OGD/R, and some axons and dendrites were lysed (Fig. 1B). We used the CCK-8 assay to assess the viability of neurons during OGD/R induction and observed that neurons were induced by OGD/R and cell viability was significantly decreased (70.36 ± 0.05%) (Fig. 1C). The results of previous studies have suggested that the reduced size of Hoechst-stained chromatin generally indicates chromatin condensation, a major feature in the nucleus of a cell undergoing apoptosis [28]. Hoechst was used to stain and assess changes in cell chromatin during OGD/R induction. During OGD/R induction, the neuronal nuclei showed size reduction and chromatin agglutinated (Fig. 1D, E). The decrease in mitochondrial membrane potential indicates that the cell is undergoing apoptosis [29, 30]. JC-1 staining showed that the mitochondrial membrane potential of neurons decreased after OGD/R induction (Fig. 1F, G). In addition, the expression of the apoptotic proteins Caspase-3, Cleaved caspase-3, and Bax increased (Fig. 1H, I). TUNEL staining results showed that after OGD/R induction, the number of TUNEL-positive cells significantly increased (36.70 ± 1.43%) relative to that of control cells (7.50 ± 0.83%) (Fig. 1J, K), indicating that the neuron OGD/R model was successfully constructed.

### m1A modification is abundant in the mRNA of primary mouse neurons

**Fig. 2:**
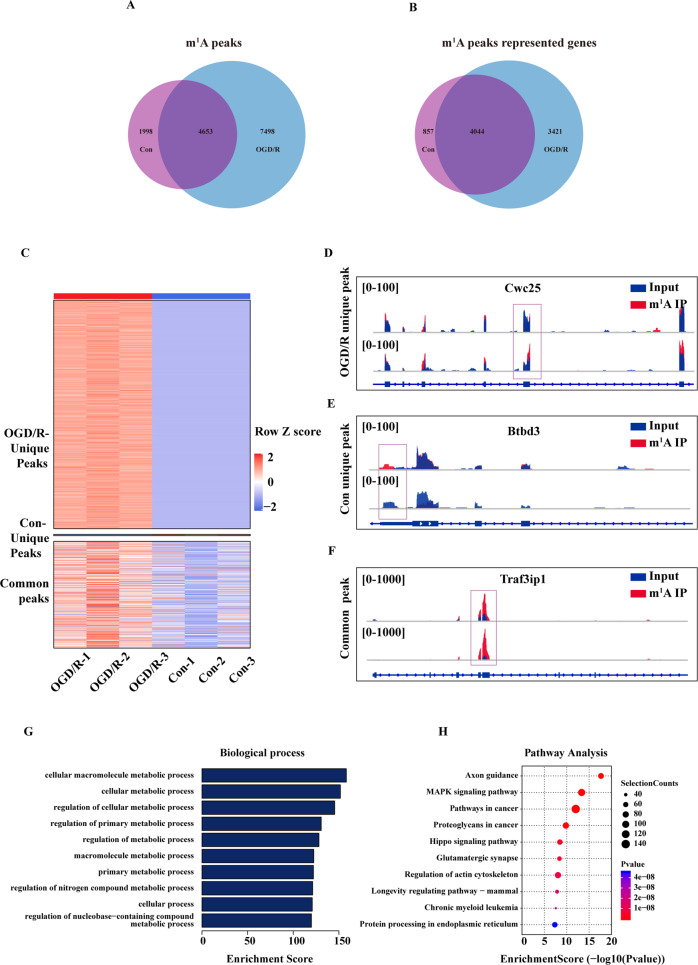
m1A modification is abundant in the mRNA of primary mouse neurons. **A** Identification of m1A peaks in mouse cortical neuron mRNA. **B** Identification of the genes mapped to the m1A peaks. **C** OGD/R-induced peaks were divided into OGD/R-specific, Con-specific, and common peaks. The fold change of each peak was normalised and is denoted with the row z score. ‘OGD/R-1’, ‘OGD/R-2’, and ‘OGD/R-3’; Con-1’, ‘Con-2’, and ‘Con-3’ denote 3 biological replicates. **D**–**F** IGV displays the representative peaks of m1A-seq. **D** OGD/R-specific peak of Cwc25. **E** Con-specific peak of Btbd3. **F** Common peak of Traf3ip1. **G** GO analysis (top 10) and **H** KEGG analysis (top 10) of the m1A-modified genes on the cortical neuron transcripts. The results of the GO and KEGG analyses with enrichment scores and *p* values.

After sequencing Q30 quality control (see Supplementary Table 3, Q30 > 80% indicates that the sequencing quality is qualified) and reference genome comparison, strict peak calling standards were used (*p* ≤ 10^−10^, fc ≥ 2) (see Supplementary Fig. S1 for biological repetition). In cortical neurons, we identified 6651 peaks corresponding to 4901 genes. After OGD/R induction, we identified 12,151 peaks corresponding to 7465 genes (Fig. 2A, B). In addition, during OGD/R induction, 7498 new peaks appeared in neurons, 1998 peaks disappeared, and 4653 common peaks corresponded to 4044 genes (Fig. 2A). We also illustrate the methylation modification peaks of the three duplicate libraries in the two groups using a heat map (Fig. 2C), which further reflected the dynamic modification of the specific m1A peaks during OGD/R induction. Subsequently, we representatively selected the methylation modification peaks in the heat map and used Integrative Genomics Viewer (IGV) to visualize the OGD/R group-specific peak Cwc25 (Fig. 2D), the Con-specific peak Btbd3 (Fig. 2E), and the shared peak between the two groups Traf3ip1 (Fig. 2F). The methylation peaks were concentrated in genes related to various metabolic pathways associated with cell components such as the mitochondria and intracellular membranes and involved in other molecular functions related to the binding of proteins and various compounds (Fig. 2G and Supplementary Fig. S2A, B). These peaks were enriched in axon guidance, MAPK, and Hippo signalling pathways (Fig. 2H). Overall, the biological processes and signalling pathways of methylation modification peak enrichment did not change significantly after OGD/R induction (Supplementary Fig. S2C–E). Similar to previous studies, we observed that m1A modification is abundant in the mRNA of cortical neurons and that it dynamically responds to OGD/R oxidative stress induction [9, 11].

### OGD/R induces different m1A methylation modification patterns in neurons

**Fig. 3:**
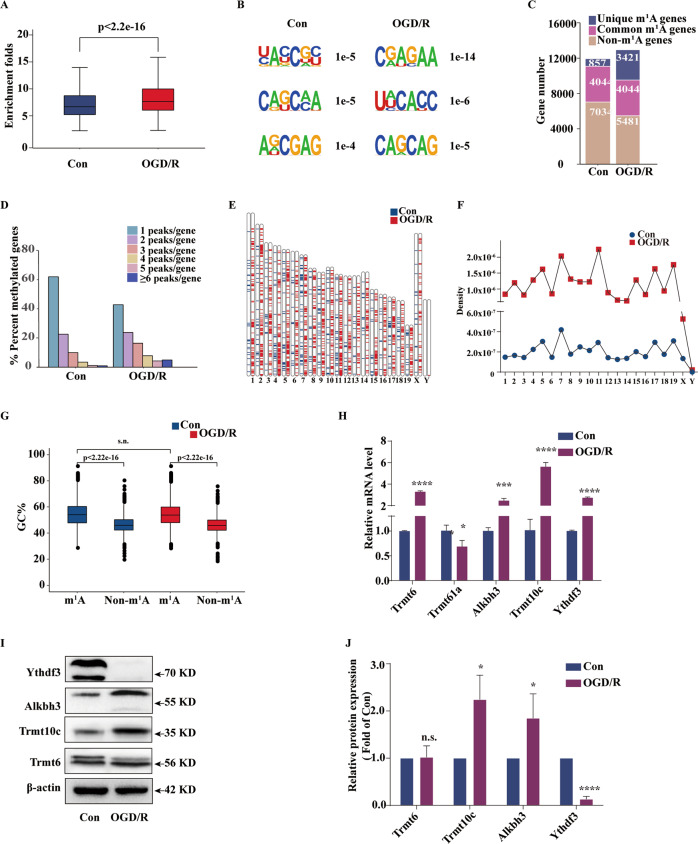
OGD/R induces different m1A methylation modification patterns in neurons. **A** Overall abundance of m1A levels in cortical neuron transcripts and after OGD/R induction. The box limits represent the upper quartile, the median, and the lower quartile. The extremes represent the maximum and minimum values (*p* < 2.2e-16, Kruskal–Wallis test). **B** Representative analysis of the modified region of the m1A transcript. **C** Transcripts are m1A modified during OGD/R induction. **D** The proportion of transcripts carrying 1, 2, 3, 4, 5, and 6 or more peaks in neurons and after OGD/R induction. **E** The distribution and **F** density of the m1A peaks on each chromosome. **G** GC content in the m1A sequence and non-m1A sequence during OGD/R induction. G + C% represents the percentage of GC dinucleotides in the sequence. **H** qRT-PCR showed changes in the expression of the methylation-related enzymes Trmt6, Trmt61a, Trmt10c, Alkbh3, and Ythdf3 in mouse cortical neurons after OGD/R induction. **I**, **J** Western blot showed changes in the expression of the methylation-related enzymes Trmt6, Trmt10c, Alkbh3, and Ythdf3 in mouse cortical neurons after OGD/R induction. Data analysis was performed using Student’s *t* test. The data are presented as the means ± SD, *n* = 3. *p < 0.05, **p < 0.01, ***p < 0.001. n.s. no significant difference.

Because mouse cortical neurons undergo dynamic regulation after OGD/R induction, we subsequently analysed the overall levels of mRNA m1A methylation in the two groups. We observed that after OGD/R induction, the overall m1A modification level was significantly increased (*p* < 2.2e-16) (Fig. 3A). Then, a motif analysis was performed on the sequence patterns unique to the methylation modification sites (Fig. 3B), the results of which confirmed that the m1A modification of cortical neurons also has a conserved sequence pattern and that the sequence pattern changes after OGD/R induction. Consistent with the transcriptome data, we observed that after OGD/R induction, a higher proportion of genes were modified by m1A compared to that observed in the control (Fig. 3C). In the analysis of the distribution of m1A peaks of methylated genes, on average, most genes (>80%) contained 1–3 peaks among the control and OGD/R induction groups, while on average, the OGD/R induction-modified genes had more m1A modification sites than the genes in the control group (Fig. 3D).

We also observed the distribution of methylation modification peaks on each chromosome (Fig. 3E) and found the most abundant peaks in both groups to be on chromosome 7 (Supplementary Fig. S3A). Density distribution analysis of methylation modification peaks on chromosomes was conducted between the two groups and showed that the peak density of methylation modification on chromosome 7 was the highest; after OGD/R induction, the density of chromosome 11 was the highest (Fig. 3F). GO analysis showed that the chromosomes of differentially modified sites (top 3) are associated with the establishment of mitochondrial localisation and microtubule-mediated effects, primarily located in the synapse, and related to the binding of RNA polymerase II transcription factors (Supplementary Fig. S3C). The differentially modified chromosomes (top 3) are enriched in the focal adhesion and Wnt pathways (Supplementary Fig. S3D). Previous studies have indicated that guanine/cytosine (GC) content is a key factor affecting the stability of the RNA structure [9]. Therefore, we also compared the GC content in the methylation modification region between the two groups but observed that the GC content in the m1A sequence did not change with OGD/R induction (Fig. 3G). This result indicates that GC content is a fixed feature of m1A methylation modification of mRNA and does not change under external pressure.

Because OGD/R can induce different m1A modification patterns in neurons, we used qRT-PCR to analyse the expression of some writer, eraser and reader genes reported thus far. We observed that the expression of the writers Trmt6/Trmt61a and Trmt10c, the eraser Alkbh3, and the reader Ythdf3 significantly changed during OGD/R induction (Fig. 3H), demonstrating that changes in these enzymes may have caused the observed differences in methylation modification patterns. We further examined the protein levels of these regulatory enzymes(Fig. 3I, J). We found significant changes in the expression levels of Trmt10c, Alkbh3 and Ythdf3, suggesting that these enzymes play a key role in regulating the level of m1A methylation and the biological function of m1A in mRNAs.

### Differential m1A modification peaks are enriched in pathways associated with neurological diseases and oxidative damage

**Fig. 4:**
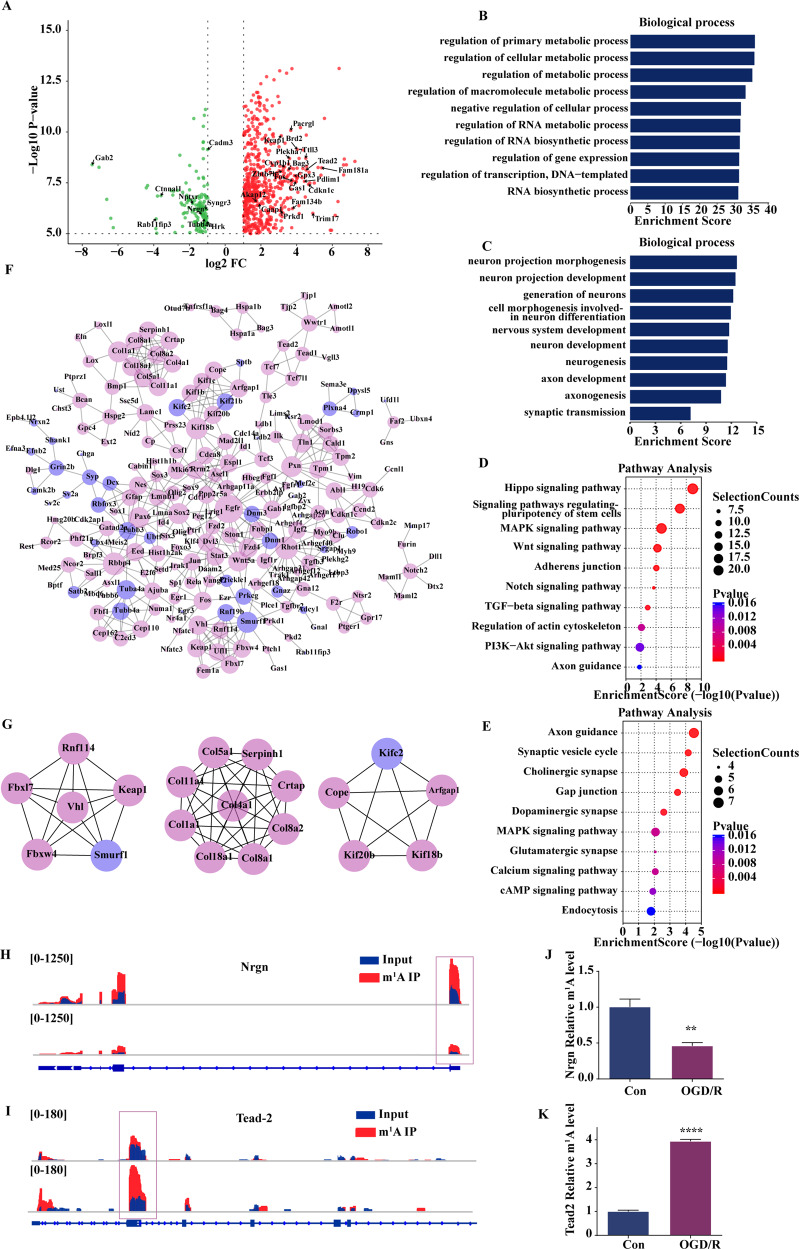
Differential m1A modification peaks are enriched in pathways associated with neurological diseases and oxidative stress damage. **A** Identification of differentially modified m1A peaks (red: upregulated, green: downregulated) (fold change ≥ 2, *p* ≤ 0.00001). **B** GO analysis of differentially modified upregulated m1A peak genes and **C** downregulated m1A peaks. **D** KEGG pathway analysis of differentially modified upregulated m1A peaks and **E** downregulated m1A peaks. **F** PPI network analysis of the differentially modified m1A peaks and **G** core genes of the top 20 interactions. The relationship of methylation regulation is colour coded. Red: upregulated genes. Blue: downregulated genes. **H**, **I** Degree of m1A methylation of the downregulated peak Nrgn during OGD/R induction. **J**, **K** The degree of m1A methylation of the Tead2 upregulated peak during OGD/R induction. GO and KEGG analyses with enrichment scores and *p* values. MeRIP-RT-PCR data were analysed using Student’s *t* tests. The data are presented as the means ± SD, *n* = 3. **p < 0.01, ****p < 0.0001.

Since neurons exhibited different m1A modification patterns during OGD/R induction, we analysed the two sets of differential modification peaks. We used criteria (fc ≥ 2, *p* ≤ 0.00001) to define differential modification peaks and identified 692 methylation peaks that were upregulated and 167 methylation sites that were downregulated (Fig. 4A). In addition, the upregulated methylation peaks are involved in the biological processes intracellular energy metabolism and RNA synthesis, decomposition and regulation (Fig. 4B) and enriched in cellular components such as the cytoskeleton and cell connections and in molecular functions such as transcription regulation (Supplementary Fig. S4A, B). The downregulated methylation peaks are associated with biological processes such as neuron regeneration and synaptic transmission (Fig. 4C) and enriched in cell components such as synaptic microtubules and molecular functions such as protein binding and GTPase activity (Supplementary Fig. S4C, D). The pathway analysis revealed that the upregulated modification peaks are closely related to axon guidance, the PI3K-AKT signalling pathway and the TGF-β signalling pathway (Fig. 4D); the downregulated peaks are related to axon guidance and the calcium ion signalling pathway (Fig. 4E). Subsequently, using Cytoscape to analyse the PPI network of the differentiated m1A-modified genes (Fig. 4F), we observed that coding collagen family members (Col1a1, Col4a1, Col5a1, Col8a1, etc.), kifc2, and smurf1 were among the top 20 key genes (Fig. 4G). This result further confirmed the key roles of differentially m1A-modified peaks in the pathways associated with neurological diseases. Furthermore, we identified some of the related genes that have been reported in the field of nervous system research (Fig. 4A) and selected a downregulated methylation modification site [Nrgn, a post-synaptic protein that regulates synapse formation and calmodulin activity [31]] and an upregulated methylation modification peak [Tead2, which plays a key role in neuron survival and the regulation of mesenchymal stem cell differentiation [32]]. Visual analysis was performed using IGV to determine the methylation level and the changes in modification intensity (Fig. 4H, I), and MeRIP-RT-PCR (Fig. 4J, K) was used to verify the methylation level to show the accuracy of our sequencing data.

### Transcript m1A modification is associated with gene expression

**Fig. 5:**
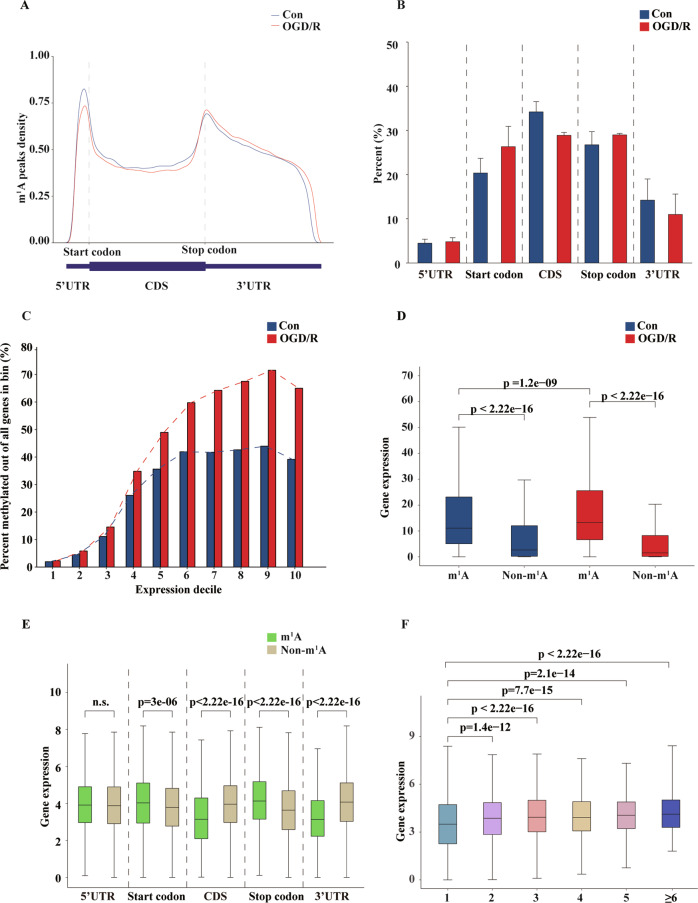
Transcript m1A modification is associated with gene expression. **A** Position distribution of m1A peaks in the transcript. mRNA is divided into 5 regions: 5′ UTR, start codon, CDS, stop codon, and 3′ UTR. According to previous studies, the regions 100-nt upstream or downstream of the start and stop codons are defined as the start codon and stop codon. **B** Percentage of m1A peaks distributed in different positions of the transcript. The data are presented as the means ± SD, *n* = 3. **C** The higher the gene expression, the higher the proportion of methylation. RNA-seq expression levels were used to separate genes into deciles, and the methylation rate of RNA-seq genes in each decile was assessed. **D** Relationship between m1A modification and gene expression. According to the m1A-seq data, each transcript was defined by whether there was a methylation-modified gene, and the expression of each gene was used to draw a box plot (*p* < 2.2e-16 and *p* < 1.2e-09, respectively; Kruskal–Wallis test). **E** m1A modifications in different regions of the transcript have different effects on gene expression. According to the area where the peaks were located and the RNA-seq expression data, a box plot was drawn (*p* = 3e-06, *p* < 1.2e-09, respectively; Student’s *t* test). **F** Effect of the number of peaks on gene expression. Classification was based on the number of peaks in the transcript and the RNA-seq expression data, which were used to draw a box plot. The box limits represent the upper quartile, the median and the lower quartile. The extremes represent the maximum and minimum values. m1A: transcripts with m1A modification; non-m1A: transcripts without m1A modification. n.s. no significant difference.

Previous studies have demonstrated that mammalian m1A mRNA is highly enriched within the 5′ UTR and near-start codons [9, 11]. Therefore, we conducted a joint comparative analysis of m1A-seq and RNA-seq data to study the m1A peaks in the transcriptome region. m1A peaks in mouse cortical neurons were enriched not only in the 5′ UTR and near the start codon but also in the 3′ UTR and near the stop codon (Fig. 5A). After OGD/R induction, the distribution of m1A enrichment peaks in the transcript region was not significantly different, indicating that the distribution of methylation peaks was not random. However, OGD/R induction caused an increase in the proportion of methylation sites in start codon and stop codon regions and a decrease in the CDS and 3′ UTR proportion (Fig. 5B). As the level of gene expression increased, the proportion of methylated genes gradually increased, and the OGD/R induction group had a higher proportion of methylated genes than the Con group (Fig. 5C). We further assessed the relationship between methylation level and gene expression and observed that m1A-modified genes have higher expression than non-m1A-modified genes, indicating that m1A modification can promote gene expression (Fig. 5D). In addition, OGD/R induction further promoted the expression of m1A-methylated parental genes relative to the control. We observed that m1A peaks in different regions of the mRNA have different effects on gene expression. The difference in the intensity of methylation modification peaks at different positions in the mRNA sequence between the two groups (Fig. 5B) may be the reason for the increased gene expression caused by OGD/R induction (Fig. 5E and Supplementary Fig. S5A, B). We also found that the number of peaks in the transcript affected gene expression (Fig. 5F and Supplementary Fig. S5C, D). OGD/R induction increased the number of methylation modification peaks in the transcripts (Fig. 3D), which is another mechanism by which gene expression may be promoted.

### Differential m1A methylation correlates positively with gene expression

**Fig. 6:**
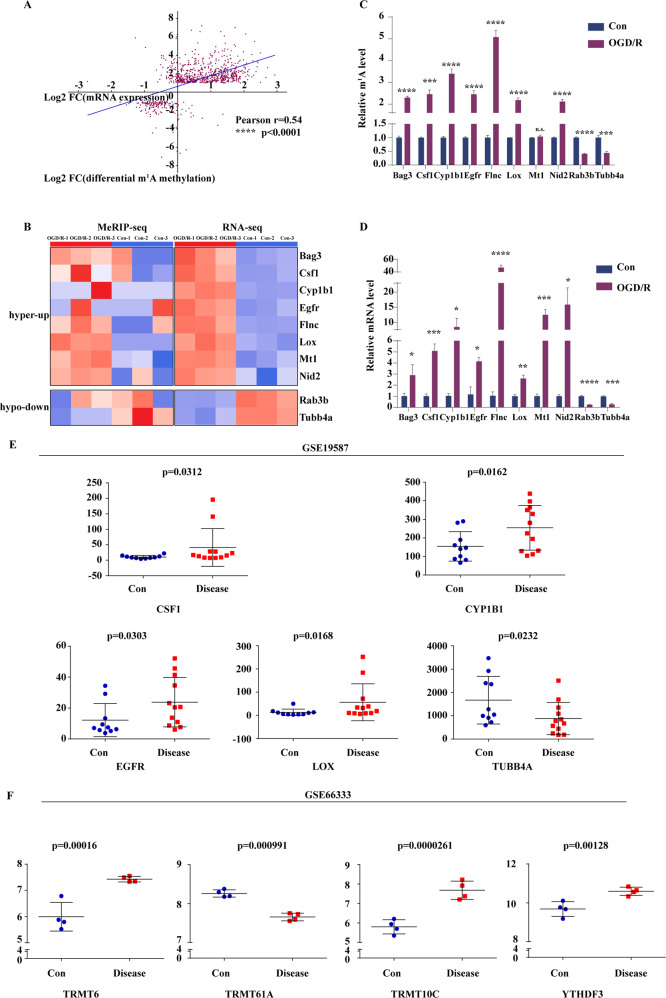
Differential m1A methylation correlates positively with gene expression. **A** The differential distribution of m1A peaks correlates positively with the gene expression. A scatter plot was drawn based on the height of the methylation peaks and the level of gene expression. cor.test was applied using the Pearson method (Pearson *r* = 0.054, *p* < 0.0001). **B** A related heat map of methylation and expression was generated for the 10 genes that exhibited a significant change in both the m1A level and mRNA transcript abundance after OGD/R induction and in primary mouse cortical neurons. **C** MeRIP-RT-PCR shows the changes in specific m1A modification levels during OGD/R induction. **D** qRT-PCR shows the changes in specific gene expression during OGD/R induction. **E** Changes in the expression of 5 specific genes in 10 control samples and 12 disease samples from the GSE19587 database. **F** Changes in the expression of 4 methylation modification regulatory enzymes in 4 control samples and 4 disease samples from the GSE19587 database. ns no significant difference. Hyper-up: highly methylated and highly expressed gene. Hypo-down: Weakly methylated and weakly expressed gene. The data are presented as the means ± SD, *n* = 3. The *p* value was calculated using Student’s t-test, *p < 0.05, **p < 0.01, ***p < 0.0001.

Since m1A methylation modification is closely associated with gene expression (Fig. 5D), and differential methylation m1A peaks are enriched in the pathophysiological processes of the nervous system, we next assessed the relationship between differential m1A methylation and gene expression by cross-analysing the m1A-seq and RNA-seq data. We observed that differentially methylated m1A peaks correlated positively with gene expression (Pearson *R* = 0.54, *p* < 0.0001) (Fig. 6A) and also defined differentially expressed genes (DEGs) (*p* ≤ 0.001, *fc* ≥ 2). Therefore, of the 303 methylation peaks with upregulated methylation levels, 224 mRNAs had upregulated expression and were designated hyper-up, while 2 mRNAs had downregulated expression and were designated as hyper-down Among the downregulated peaks, 14 mRNAs had upregulated expression and were designated hypo-up, while 1 mRNA had downregulated expression and was designated hypo-down. Nid2, Gfap, Dlk1, Flnc, Lox, Ajuba, Vasn, Arsi, Vim, and Serinc2 were the top ten genes with the most significant upregulated differential expression. Erdr1, Nrgn, Rab3b, Kifc2, Sema3e, Syp, Dnm1, Tubb4a, Tenm1, and Adcy1 were the top ten genes with the most significant downregulated differential expression (Supplementary Tables 4 and 5). Notably, we observed that most of the peaks with upregulated methylation were associated with highly expressed genes, while those with downregulated methylation were associated with weakly expressed genes. We selected previously reported genes that were of interest to us (Table 1), including some genes with high expression and high methylation (Bag3, Csf1, Cyp1b1, Egfr, Flnc, Lox, Mt1, and Nid2) and some with low methylation and low expression (Rab3b, Tubb4a), and generated heat maps to assess the relationship between the two databases (Fig. 6B). In addition, we selected and compared the MeRIP-RT-PCR (Fig. 6C) and qRT-PCR (Fig. 6D) results to verify the accuracy of our findings, and the results showed the reliability of the sequencing data and our conclusions. Due to the strong correlation of these genes with apoptosis, we also examined the expression levels of these proteins in neurons after OGD/R induction. We confirmed the high protein level expression of the hypermethylation modifier genes Csf1 and the low protein level expression of the hypomethylation modifier genes Rab3b and Tubb4A (Supplementary Fig. S6A, B). We strengthen the correspondence between m1A modifications in mRNAs and methylation.

**Table 1:** Ten genes exhibiting a significant change in both m1A levels and mRNA transcript abundance in OGD/R induction and mouse primary cortical neurons.

Gene name	Pattern	Peak region	Peak start	Peak end	m1A Foldchange	m1A *P* value	mRNA Foldchange	mRNA *P* value
Bag3	Hyper-up	Start codon	128523582	128524044	11.33687943	7.3601E-09	3.96664	0.00005
Csf1	Hyper-up	3′ UTR	107741741	107742540	3.2639425	4.4419E-08	3.67096	0.00005
Cyp1b1	Hyper-up	CDS	79710381	79710681	9.801282051	5.2549E-09	2.89032	0.00005
Egfr	Hyper-up	3′ UTR	16912401	16912760	3.835978836	1.4166E-07	3.15316	0.00005
Flnc	Hyper-up	3′ UTR	29461301	29461780	19.20261438	1.7495E-08	5.64498	0.00005
Lox	Hyper-up	CDS	52528716	52529120	9.289340102	1.4001E-08	5.37706	0.00005
Mt1	Hyper-up	Stop codon	94179822	94180327	3.137704206	1.0047E-08	4.72783	0.00005
Nid2	Hyper-up	CDS	19768209	19768640	6.389144434	2.183E-11	6.57613	0.03695
Rab3b	Hypo-down	Stop codon	108940641	108941040	2.614019779	7.4005E-07	−2.91442	0.00005
Tubb4a	Hypo-down	Stop codon	57080065	57081080	2.284453578	1.8945E-06	−2.27046	0.00005

Finally, we selected human tissue samples from individuals with PD (GSE19587) from the Gene Expression Omnibus (GEO) database to analyse the selected DEGs and observed that the gene expression of Csf1, Cyp1b1, Egfr, Lox, and Tubb4a was consistent with that observed in our present study (Fig. 6E). We also selected brain tissue samples from individuals with AD (GSE66333) to analyse the differential methylation modification regulatory enzymes and observed that the gene expression of Trmt6, Trmt61A, TRMT10C, and YTHDF3 was consistent with that observed in our present study (Fig. 6F). This analysis proved the important role of m1A mRNA methylation modification in nervous system damage.

## Discussion

Studies in recent years have proven that abundant m1A modifications exist on the mRNA of eukaryotic cells. m1A modifications, which are enriched in the 5′ UTR and near the start codon, can improve protein translation efficiency [9–11], and m1A modifications of mRNA are dynamically by m1A writers, erasers, and readers and external pressure [9, 11, 33–36]. In addition, modification of the m1A transcript has been shown to regulate mRNA metabolism during cellular stress [37]. However, the abundance, enrichment location, and function of mRNA m1A and OGD/R induction in primary mouse cortical neurons are unclear.

Similar to previous studies, the results of our present study confirmed some typical modification characteristics of m1A methylation in mRNA. m1A modification is abundant in the mRNA of neurons, and these peaks were concentrated in the 5′ UTR, near the start codon, and in the 3′ UTR, near the stop codon, showing a “bimodal” distribution (Fig. 5A). After OGD/R induction, the enrichment positions of the methylation peaks did not change. This phenomenon contrasts with that described in previous reports. In human tumour cell lines, mouse embryonic stem cells, and primary mouse embryonic fibroblasts [9], m1A is highly enriched in the 5′ UTR of mRNA and in the CDS region in petunia [13], indicating that the m1A methylation modification pattern is different in different species and even in different tissues from the same species. This phenomenon may be due to the effects of m1A methylation regulatory enzymes (Trmt6, Trmt61a, Trmt10c, Alkbh3, and Ythdf3) [9, 11, 34–36], which can significantly change the m1A modification level and modification pattern, which is primarily manifested by an increase in the number of peaks and the gene methylation ratio, a change in representative modification sequences, and a difference in the distribution difference of chromosome density. GO and KEGG signal pathway analyses revealed that m1A in neurons is associate with metabolism, axon guidance and so on. We analysed the differential methylation modification peaks that were enriched in the nervous system and OGD/R model-related pathways, such as neuron differentiation, electrical signal transduction, axon regeneration, synaptic connections, the cytoskeleton, energy generation and gene regulation (Fig. 4B–E). Subsequently, we selected Tead2 and Ngrn, two genes that play important roles in the nervous system, and constructed a PPI network to further confirm that mRNA m1A methylation modification is of great significance to neuronal damage.

Many studies have shown a correlation between the post-transcriptional modification of genes and gene expression. m6A modification in clear cell renal cell carcinoma (ccRCC) can promote the expression of cancer-related genes [38], and the methylation level of m6A/m transcripts correlates negatively with changes in RNA [39]. We observed that m1A modification may promote gene expression and that the enrichment position and average number of methylation modification peaks in mRNA can affect gene expression. One study confirmed that m1A located in the first and second positions of the 5′ UTR in the transcript can promote protein translation, while m1A located in the CDS can inhibit translation [10]. Based on our results, m1A peaks located in the start and stop codons promote gene expression, whereas those located in the CDS and 3′ UTR inhibit gene expression (Fig. 5E). This finding indicates that m1A peaks at different positions in the transcript have different effects on transcription and translation. This change has a substantial impact on gene expression, considering the proportion of genes with methylation modification, although this accounts for a small proportion of changes. A positive correlation between m1A modification and gene expression was observed (Fig. 6A), and we selected genes related to neurological diseases (Bag3, Csf1, Cyp1b1, Egfr, Flnc, Lox, Mt1, Nid2, Rab3b, and Tubb4a) for analysis, with MeRIP-RT-PCR and qRT-PCR performed to verify this phenomenon. It is worth emphasising that these genes are also strongly associated with apoptosis (supplementary Fig. S6). However, studies have also shown that the human YTH domain family 2 (YTHDF2) reader protein can regulate mRNA degradation, proving that post-transcriptional modifications can affect the stability of transcripts [30]. Research has also shown that the TRMT6/61 A methyltransferase is involved in granulation and safeguarding of mRNAs during stress [37]. Therefore, whether this phenomenon is due to the direct increase in gene expression caused by the modification of m1A or the loss of mRNA expression caused by the change in the level of the enzyme is currently unclear.

The relationship of m1A modification in mRNAs to apoptosis is currently unclear. A gastrointestinal tumor-based bioinformatics analysis revealed that the canonical apoptosis-related pathways ErbB and mTOR signalling were identified as pathways regulated by m1A-related enzymes. After ALKBH3 gene knockout, the expression of the identified Hub genes ErbB2 and AKT1S1 were decreased, indicating that m1A modification in mRNA is involved in the regulation of apoptosis [40]. In addition, tRNA m1A demethylase ALKBH3 increases sensitivity of tRNA to angiogenin (ANG) cleavage, leading to the formation of tRNA-derived small RNAs (tDRs), and ALKBH3-generated tDRs triggers the ribosome assembly and interacts with Cyt c to prevent cell death [41]. In our study, we also found high expression of Alkbh3 during OGD/R induction (Fig. 3G, I), suggesting that Alkbh3 regulates the m1A modification in neuronal mRNA involved in the process of neuronal apoptosis. We found that m1A methylation may promote gene expression (Fig. 5), and additional studies showed that all 10 genes validated were closely related to apoptosis (Fig. 6 and supplementary Fig. S6). In conclusion, our study provides a potential idea that m1A modification might promote neuronal apoptosis. However, high-level validation experiments are still lacking. Therefore, higher-level studies such as the use of genetic programmes to intervene m1A methylation-regulating enzymes, and further observe changes in neuronal apoptosis through such methods are more important.

Recent studies have shown that neurodegenerative diseases such as AD and PD are associated with cerebral IRI [25, 42]. The immunoreactivity of tau protein in neuronal cells increases after cerebral IRI, causing neuronal oxidative stress, leading to enhanced autophagy and apoptosis and accelerating the pathological process of AD [25]. The progressive loss of neurons is a key factor in the neurological dysfunction of AD and PD patients [43]. Our results also show that neurons undergo apoptosis during OGD/R induction. In addition, post-transcriptional modification of RNA has been shown to be closely related to the occurrence and development of neurodegenerative diseases. m6A controls RNA stability, splicing, translation, and transport, plays an important role in learning and memory [44]. Studies have shown that in AD mice, the expression of the m6A methyltransferase METTLE3 increases, while that of the m6A demethylase FTO decreases, and it has been confirmed that m6A methylation of mRNA promotes the development of AD [45]. Other studies have also demonstrated that the m6A modification of RNA plays a key role in the development of human AD and PD [46]. Our results reveal the potential role of transcript m1A modification in AD and PD (Fig. 6E, F). Neurons are induced by OGD/R, and differential m1A modification participates in the regulation of differential gene expression. Among the highly DEGs identified, studies have confirmed that the expression of the Nid2, Gfap, Dlk1, Lox, Vim, and Syp genes is related to AD or PD (Supplementary Tables 4 and 5) [47–52]. Our results also confirmed the important role of neuronal IRI in neurodegenerative diseases and the potential role of m1A modification in transcripts.

Since the m1A modification of mRNA in mice and humans is highly conserved [9] and m1A methylation is closely related to neurological diseases, we also utilized human tissue samples from the GEO database to verify the mRNA features of m1A modification (Fig. 6E, F). In brainstem tissues from humans with PD [53], we observed that the gene expression of CSF1, CYP1B1, EGFR, LOX and TUBB4A was consistent with that observed in our validation study. In frontal cortex tissues from humans with AD [54], the gene expression of TRMT6, TRMT61A, TRMT10C, and YTHDF3 was consistent with that observed in our verification study, indicating that mRNA m1A methylation modification also plays a key role in human neurological diseases.

However, due to the characteristics of m1A [m1A can rearrange to m6A under alkaline conditions (Dimroth rearrangement) [55], there is controversy regarding the abundance of m1A in the transcript [56]. Li et al. [11] identified 901 high-confidence m1A peaks from the transcriptome of HEK293T cells [11], and Dominissini et al. [9] identified 3940 peaks in HepG2 cells using similar measurement methods. Subsequently, these two independent research groups developed transcriptome-wide approaches (m1A-MAP and m1A-seq-TGIRT) to map m1A methylomes at single-base resolution [10, 35]. Based on these research methods, Li et al. [10] identified 473 sites in mRNAs and long non-coding RNAs, while Safra et al. [35] identified only 15 m1A sites. However, some researchers have observed that the m1A/A ratio in human genes is ~0.02% [9, 11], indicating that there are abundant m1A modification sites in the human genome, not only the sites detected by Safra et al. [56]. Therefore, some researchers have proposed that the sensitivity of m1A-seq-TGIRT is limited (e.g., severe repeated reading, rRNA contamination) [56, 57]. In the present study, we identified 4901 peaks (535 repetitive peaks were identified in the three biological samples, see Supplementary Fig. S1A) in mouse cortical neurons, proving the prevalence of m1A modification in neuronal transcripts. In addition, further studies using new methods (m1A-IP-seq and m1A-quant-seq) to more effectively identify the evolution of the reverse transcription of m1A have also reported hundreds of m1A sites, further confirming that m1A is widespread in mRNA [56, 58]. Therefore, more specific antibodies and increased recognition depth of m1A sites are needed to assess the abundance of m1A modifications in transcripts.

## Conclusions

In summary, we performed transcriptome-wide profiling of m1A in the mRNA of mouse cortical neurons and an OGD/R induction model, and the findings provide a better understanding of neuronal mRNA post-transcriptional modification and confirm the significance of mRNA m1A in neurological diseases. The results of the present study offer new ideas for understanding the mechanism of neuronal oxidative damage and nerve damage interventions.

## Materials and methods

### Ethics statement

The C57BL/6 mice used in the present study were purchased from the Laboratory Animal Centre, Academy of Military Medical Science (Beijing, China). All experiments were performed in adherence with the National Institutes of Health Guidelines on Laboratory Animals and were approved by the Ethics Committee of Qilu Hospital, Shandong University (Jinan, Shandong, China; DWLL-2021-061).

### Isolation and culture of cortical neurons

Cortical neurons were isolated and cultivated as described in our previous studies [59, 60]. Primary cortical neurons were derived from embryonic 17th C57BL/6 mice. After euthanizing pregnant mice, the mice were sterilised in an ice bath containing 75% alcohol for 5 min. Then, the abdominal cavity was opened in a sterile environment in Dulbecco’s modified Eagle’s medium (DMEM, Gibco, 31053028). Under a stereomicroscope (Olympus, Tokyo, Japan), the fetal cerebral cortical tissue was dissected and cut into small pieces (1 mm^3^), followed by incubation in 2 mg/ml papain (Worthington, LS03126) and 20 units/ml DNase for 15 min at 37 °C (Sigma-Aldrich, P4762). Subsequently, the cells were dispersed in DMEM containing 10% fetal bovine serum (Gibco, 10099-141). The cell density was adjusted to 50,000 cells/cm^2^ by counting, and the cells were plated in a Petri dish coated with 0.01% poly-D-lysine (Sigma-Aldrich, P4707) in a 37 °C incubator under an atmosphere with 5% CO_2_. After 4 h, the previous medium was replaced with neurobasal medium (Gibco, 21103049) containing 1% B27 additive (Thermo Scientific, 17504044), 2 mM L-glutamine (Gibco, 25030081) and 1% penicillin/streptomycin (Thermo Scientific, 15140148) at 37 °C under an atmosphere with 5% CO_2_ for cultivation in an incubator, with the culture medium replaced every three days.

### Immunofluorescence

On the 6th day of cell culture, the cells were fixed with 4% paraformaldehyde for 10–15 min, and 0.1% Triton X-100 was loaded into each well to react for 5 min. To reduce nonspecific binding, 10% goat serum (Solarbio, SL038) was added for 2 h at room temperature. Then the primary antibody β-III tubulin (Abcam, ab78078, 1:400) was added to the anti-labelled neurons and axons, and incubated at 4°C overnight. Then the corresponding secondary antibodies were incubated at room temperature for 1 h. After being washed with PBS, the cells were incubated with DAPI (Beyotime, C1002) or Hoechst 33342 (Abcam, ab228551) to label nuclei for 10 min, and images were obtained under a fluorescence microscope or confocal microscope. Under the microscope, the ratio of β-III tubulin-positive to DAPI cells was counted in three random fields to determine the purity of the extracted neurons.

### OGD/R model preparation

OGD/R model construction was performed as previously described by our lab and others [60, 61]. On the 6th day of cell culture, the neurobasal medium was replaced with glucose-free DMEM (Gibco, 31053028) before washing the cells with PBS following the manufacturer’s instructions (bioMerieux, 45534), after which the neurons were placed under hypoxic conditions for 1.5 h. Then, the glucose-free DMEM was replaced with neurobasal medium for 24 h (37 °C, 5% CO_2_) to simulate the reperfusion state.

### TUNEL assay

According to the instructions provided by the manufacturer of a terminal deoxynucleotidyl transferase dUTP nick end labelling (TUNEL) assay kit (Roche, 11684817910), the cells were fixed with 4% paraformaldehyde for 15 min after the cell culture medium was discarded during OGD/R induction. Then, the cells were treated with 0.1% Triton X-100 for 5 min and washed with PBS. Then, the prepared TUNEL reaction solution, containing 50 µl of enzyme solution and 450 µl of label solution mixture, was added to the cells. Subsequently, the solutions were allowed to react for 1 h before being mixed with 100 µl of a DAPI-containing nuclear reaction solution for 10 min. Finally, the cells were observed under a fluorescence microscope. Three fields of vision were randomly selected in each well, and the percentage of positive cells was obtained with the following formula: number of blue and green double-labelled cells/number of blue-labelled cells × 100%. The experiment was performed with three independent biological replicates.

### Western blot

After the cells were washed with PBS, RIPA lysis buffer (Solarbio, R0020) containing protease inhibitors was added to fully lyse the cells at 4 °C for 30 min, which was followed by centrifugation at 13,000 × *g* at 4 °C for 10 min. Then, the supernatant was collected, and a PierceTM BCA Protein Assay Kit (Thermo Scientific, 23225) was used to determine the protein concentration. Finally, the protein was denatured at 100 °C for 10 min. Subsequently, 25 µg of protein was separated by 12% SDS-PAGE and transferred to PVDF membranes, which were blocked with 5% skim milk for 1 h and then incubated with primary antibodies on a shaker at 4 °C overnight. The antibodies used in the present study included those against β-actin (MBL International, JM-3598R-100, 1:10000), caspase-3 and cleaved caspase-3 (CST, 9662, 1:1000), Bax (CST, 2772, 1:1000), Trmt6 (proteintech, 16727-1-AP, 1:500), Trmt10c (proteintech, 29087-1-AP, 1:500), Alkbh3 (CST, 87620 S, 1:1000), Ythdf3 (Abcam, ab220161, 1:1000), Bag3 (Abcam, ab92309, 1:1000), M-CSF (Abcam, ab233387, 1:1000), Egfr (Abcam, ab52894, 1:1000), Lox-1 (Abcam, ab174316, 1:1000), Nid2 (Abcam, ab131279, 1:1000), Rab3b (Abcam, ab177949, 1:1000) and Tubb4a (Abcam, ab179509, 1:1000) which were diluted in Tris-buffered saline containing 0.1% Tween (TBST). After washing the membranes in TBST solution, the membranes were incubated with horseradish peroxidase-linked anti-rabbit IgG (CST, 7074, 1:10,000) for 1 h. Finally, a horseradish peroxidase mixture was added to expose the bands on an exposure instrument, and ImageJ was used for greyscale value analysis.

### RNA extraction and qRT-PCR

Following the manufacturer’s instructions, total RNA was immediately extracted with TRIzol when the OGD/R induction process was completed, and chloroform extraction and isopropanol purification were performed to separate and precipitate the RNA. RNA concentration was evaluated with a NanoDrop ND-1000 instrument. The ratio of OD260/OD280 is an indicator of RNA purity, and when the OD260/OD280 value ranged from 1.8 to 2.1, RNA was considered to be non-contaminated (see Supplementary Table 1). RNA integrity was assessed by agarose gel electrophoresis. Subsequently, the RNA was reverse transcribed according to the requirements of a RevertAid First Strand cDNA Synthesis Kit (Thermo Scientific, K1622). The RNA concentration was adjusted using the UltraSYBR Mixture (Cwbio, CW0957M) reaction system for detection on a Roche real-time quantitative PCR instrument. Information on the primers used for qRT-PCR is shown in Table 2.

**Table 2:** Primer information of qRT-PCR and MeRIP-RT-PCR.

qRT-PCR primer		
Gene name	Forward	Reverse
Bag3	ATTCAGGTCACCCGTCAGAG	TTTCGGGTTGGGTAACAGGT
Csf1	GTGTCAGAACACTGTAGCCAC	TCAAAGGCAATCTGGCATGAAG
Cyp1b1	CAGTCTGGCGTTCGGTCAC	GCTGCGTTGGATCGAGGAA
Egfr	GCCATCTGGGCCAAAGATACC	GTCTTCGCATGAATAGGCCAAT
Flnc	GAAGGCCAACATCCGAGACAA	AGGGCGAGTAAGGGATCTCAT
Lox	ACTTCCAGTACGGTCTCCCG	GCAGCGCATCTCAGGTTGT
Mt1	AAGAGTGAGTTGGGACACCTT	CGAGACAATACAATGGCCTCC
Nid2	CTCTTTCCTTACGGGGAGTCG	GGCATCGTAGAAACGCAGG
Rab3b	TCAGATTAAGACCTACTCCTGGG	AGTTGGGACCACTCTTTCCTC
Tubb4a	CTATGTTCCCAGAGCCGTGC	CAGGACGGCATCCACTAACT
Trmt6	GTGGTGCTGAAGCGAGAAGAT	CTATGGCCGATGGCGTTATCC
Trmt61a	CGCACGCAGATCCTCTACTC	GGAACTCTACTGTGTGTAGGTGG
Trmt10c	GGAAGCCGTGCTGTAGGA	AGCTGCTCAGGAGGGGAT
Alkbh3	GCCAGGTAGCCATCCCTT	AGGGGAAGCTGGCTGAGT
Ythdf3	GCTGCGGTGACGAAAACT	TGCTAACAGGGGGCACAT
Gapdh	AGGTCGGTGTGAACGGATTTG	TGTAGACCATGTAGTTGAGGTCA
**MeRIP-RT-PCR primer**		
**Gene name**	**Forward**	**Reverse**
Csf1	GGCTTGCTTGGCTAGAGATG	TGTCCTGTCACAAGCTCTGG
Cyp1b1	GCTGGATCAAAGTCCTCTGG	TTCTCCAGCTTTTTGCCTGT
Egfr	ATGTCCCTGGCACCTAACAC	GGGTTCGAATGTGGAATCAT
Flnc	CCGTGCCTAAGGACTCTGTC	ACATCACATGCTGCTTGCTC
Lox	AAACCAGCTTGGAACCAGTG	TTCTTCTGCTGCGTGACAAC
Mt1	AGTGAGTTGGGACACCTTGG	TGTCCCCAACAGAGAAGACC
Nid2	CACCGAGGACAGTTTCCATT	CCAGTTACCAGGTGCTGGAT
Nrgn	TCTTTCCTAGCCCCAGGTTT	CAACCACCAAGTCCTTTCGT
Rab3b	TGCAGCAGAACTGCTCTTGT	GGATTGGGGAATGGACAGTA
Tead2	CTGGACAGGTAGCGAGGAAG	CATCTTGCCCTCATCTGACA
Bag3	GGACCCTAACCCAGCATGAG	TTGTGGTCCACGAAGAAGGG
Tubb4a	TGCAGGGGTGTGATGCTTAG	CAGGATGCCACTGCTGAAGA

### MeRIP library preparation and sequencing

We used a commercially available m1A antibody (MBL International, D345-3,) to co-immunoprecipitate methylated mRNA fragments according to previously described protocols [11, 13]. After the neurons were induced by OGD/R, TRIzol was used to immediately extract total RNA. After concentration and quality testing, total RNA was rRNA depleted with Ribo-zero (Illumina, 20040526) and then fragmented using RNA fragmentation reagents (Thermo Scientific, AM8740). Fragmented RNA was then incubated with the anti-m1A polyclonal antibody (MBL International, D345-3) in IPP buffer for two hours at 4 °C. Subsequently, the mixture was immunoprecipitated by incubation with Protein A beads (Thermo Scientific, 21348) at 4 °C for an additional 2 h. Bound RNA was eluted from the beads with N ^1^-methyladenosine (BERRY & ASSOCIATES, PR3732) in IPP buffer and extracted with TRIzol reagent according to the manufacturer’s instructions. The m1A-to-m6A rearrangement was induced by incubating the input and immunoprecipitation fragments in alkaline buffer (50 mM Na_2_CO_3_ and 2 mM EDTA, pH 10.4) for 1 h. The purified RNA was used to generate the RNA-seq library with the NEBNext® Ultra™ RNA Library Prep Kit (NEB, E7530L) (Supplementary Table 2) and was sequenced on an Illumina HiSeq 4000 sequencer, with cutadapt (v1.9.3) and HISAT used for quality control. MACS-2 and diffReps software were used to identify methylation and differential methylation sites (fold change (fc) ≥ 2, *p* value ≤ 0.0001) in the RNA library compared to a reference genome, respectively.

### Bioinformatics analysis

Gene ontology (GO) analysis was performed using the Database for Annotation, Visualization and Integrated Discovery (DAVID) Bioinformatics Resources web tools (https://david.ncifcrf.gov/) for annotation and clustering. In addition, Kyoto Encyclopedia of Genes and Genomes (KEGG) analysis was also performed with the web tools database (https://www.genome.jp/kegg/) and with data described elsewhere [62]. Cytoscape (3.7.0) was used to analyse the protein-protein interaction (PPI) network. MetaplotR (R language software package) was used to draw metagene plots. Metagene plots are density maps or histograms of target sites (such as protein binding sites or RNA modifications) of a simplified transcript model containing the 5′ UTR, coding sequence and 3′ UTR. Subsequently, 5′ UTR, 3′ UTR, coding sequence (CDS), stop codon and start codon annotation was performed for each group of peaks and bar graphs were generated based on the annotation results.

### MeRIP-RT-PCR

Anti-RNA methylation-modified monoclonal antibodies and the A/G magnetic beads method were used as previously described [10–12]. First, the RNA was divided into 100-nt fragments, and the appropriate amount of fragmented RNA was saved as input. Then, the m1A antibody and A/G magnetic beads were added sequentially and incubated for 2 h, with an MeRIP sample subsequently obtained after elution and purification. Finally, RT-PCR was performed on both the fragmented RNA and the corresponding input RNA, and all sample analyses were performed in triplicate.

### Cell viability

A CCK-8 analysis kit (Beyotime, C0038) was used following the manufacturer’s instructions to determine the viability of the cells in the 96-well plate after the cells were induced by OGD/R. To this end, 10 μl of CCK-8 and 90 μl of neurobasal medium were mixed and added to each well, and the cells were then incubated at 37 °C for 4 h in the dark. Subsequently, the absorbance at 450 nm was measured using a microplate reader. Each sample was assayed in triplicate.

### Mitochondrial membrane potential measurement

The mitochondrial membrane potential was determined with a mitochondrial membrane potential assay kit with JC-1 (Beyotime, C0026) according to the manufacturer’s instructions. In brief, neurons were induced by OGD/R, after which the cells were washed twice with PBS. Then, we added 1 ml of JC-1 staining working solution for 30 min, after which the cells were washed twice with JC-1 staining buffer. Finally, we observed the cells through a fluorescence microscope.

### Statistical analysis

For each assay, three independent biological and technical replicates were performed. The data are presented as the means ± standard deviation (SD) of independent experiments. GraphPad Prism 6 (version) was used to analyse and compare all groups using Student’s t-test, unless otherwise noted. *P* < 0.05 was considered to indicate a significant difference.

## Supplementary information

Supplementary Tables

Supplementary Figure 1

Supplementary Figure 2

Supplementary Figure 3

Supplementary Figure 4

Supplementary Figure 5

Supplementary Figure 6

Supplementary Figure and Table Legends

Original Data File

## Data Availability

Sequencing data are available upon request to the corresponding author. Some data that support the findings of our study are openly available in the Gene Expression Omnibus (GSE19587 and GSE66333) at https://www.ncbi.nlm.nih.gov/geo/.
