# Supplementary material for: N1-Methyladenosine modification of mRNA regulates neuronal gene expression and oxygen glucose deprivation/reoxygenation induction
Source: Cell Death Discov. 2023 May 12;9:159. doi: 10.1038/s41420-023-01458-2 (PMC10182019; doi:10.1038/s41420-023-01458-2)
Supplement: Supplementary file 1 — Supplementary Tables [file 41420_2023_1458_MOESM1_ESM.docx]

**Supplementary Table 1**

| Sample name | OD260/280 Ratio | RNA concentration (μg/μl) |
| --- | --- | --- |
| Con-1 | 1.81 | 1568.69 |
| Con-2 | 1.82 | 1266.46 |
| Con-3 | 1.84 | 1229.37 |
| OGD/R-1 | 1.85 | 771.36 |
| OGD/R-1 | 1.81 | 769.53 |
| OGD/R-1 | 1.87 | 657.75 |

**Supplementary Table 2**

| Sample name | Size (bp) | Concentration (ng/µl) |
| --- | --- | --- |
| Con-1. IP | 275 | 7.08 |
| Con-2. IP | 274 | 7.66 |
| Con-3. IP | 275 | 5.73 |
| OGD/R-1. IP | 278 | 3.04 |
| OGD/R-1. IP | 274 | 6.52 |
| OGD/R-1. IP | 277 | 8.16 |
| Con-1. Input | 317 | 3.86 |
| Con-2. Input | 307 | 4.19 |
| Con-3. Input | 300 | 3.36 |
| OGD/R-1. Input | 325 | 2.69 |
| OGD/R-1. Input | 306 | 5.63 |
| OGD/R-1. Input | 299 | 4.86 |

**Supplementary Table 3**

| Sample name | Q30 |
| --- | --- |
| Con-1. IP | 86.19% |
| Con-2. IP | 85.78% |
| Con-3. IP | 84.09% |
| OGD/R-1. IP | 87.18% |
| OGD/R-1. IP | 86.72% |
| OGD/R-1. IP | 86.98% |
| Con-1. Input | 89.84% |
| Con-2. Input | 89.81% |
| Con-3. Input | 90.86% |
| OGD/R-1. Input | 89.01% |
| OGD/R-1. Input | 89.04% |
| OGD/R-1. Input | 89.05% |

**Supplementary Table 4**

| Gene name | chromosome | Peak Start | Peak end | m1A Foldchage | m1A P-value | m1A Regulation | Strand | mRNA Foldchage | mRNA P-value | mRNA Regulation |
| --- | --- | --- | --- | --- | --- | --- | --- | --- | --- | --- |
| Nid2 | chr14 | 19768209 | 19768640 | 6.389144 | 2.18E-11 | up | + | 6.5761353825000004 | 0.03695 | up |
| Gfap | chr11 | 102888201 | 1.03E+08 | 3.013639 | 2.08E-10 | up | - | 6.3611164609799999 | 0.00005 | up |
| Dlk1 | chr12 | 109459941 | 1.09E+08 | 2.09198 | 2.25E-08 | up | + | 5.6712627578100001 | 0.00005 | up |
| Flnc | chr6 | 29461301 | 29461780 | 19.20261 | 1.75E-08 | up | + | 5.6449859882300002 | 0.00005 | up |
| Lox | chr18 | 52528716 | 52529120 | 9.28934 | 1.4E-08 | up | - | 5.3770690567099999 | 0.00005 | up |
| Ajuba | chr14 | 54576921 | 54577540 | 10.29484 | 5.03E-13 | up | - | 5.2832658241599999 | 0.00005 | up |
| Vasn | chr16 | 4649081 | 4649680 | 4.69932 | 1.45E-10 | up | + | 5.1700204961300003 | 0.0001 | up |
| Arsi | chr18 | 60911779 | 60912180 | 45.60714 | 2.14E-11 | up | + | 5.0146186797799999 | 0.00005 | up |
| Vim | chr2 | 13574061 | 13574780 | 3.578619 | 4.35E-08 | up | + | 4.8962254858899996 | 0.00005 | up |
| Serinc2 | chr4 | 130253494 | 1.3E+08 | 10.63248 | 1.61E-08 | up | - | 4.8428959269799998 | 0.00005 | up |

**Supplementary Table 5**

| Gene name | chromosome | Peak Start | Peak end | m1A Foldchage | m1A P-value | m1A Regulation | Strand | mRNA Foldchage | mRNA P-value | mRNA Regulation |
| --- | --- | --- | --- | --- | --- | --- | --- | --- | --- | --- |
| Erdr1 | chrY | 90793295 | 90793680 | 2.204964 | 5.05E-08 | up | + | -6.57294539567 | 0.0081 | down |
| Nrgn | chr9 | 37552536 | 37552860 | 2.538851 | 1.1E-06 | down | - | -3.32208733249 | 0.00005 | down |
| Rab3b | chr4 | 108940641 | 1.09E+08 | 2.61402 | 7.4E-07 | down | + | -2.9144224229 | 0.00005 | down |
| Kifc2 | chr15 | 76667341 | 76667980 | 2.372233 | 1.26E-06 | down | + | -2.76117054612 | 0.00005 | down |
| Sema3e | chr5 | 14252541 | 14252860 | 60 | 6.83E-06 | up | + | -2.41645143454 | 0.00005 | down |
| Syp | chrX | 7652341 | 7653256 | 3.126767 | 6.65E-06 | down | + | -2.3552823178 | 0.00005 | down |
| Dnm1 | chr2 | 32308741 | 32309540 | 2.289468 | 2.6E-07 | down | - | -2.32749811601 | 0.00005 | down |
| Tubb4a | chr17 | 57080065 | 57081080 | 2.284454 | 1.89E-06 | down | - | -2.27046846655 | 0.00005 | down |
| Tenm1 | chrX | 43428561 | 43429126 | 3.181109 | 6.24E-07 | down | - | -2.20917851209 | 0.00005 | down |
| Adcy1 | chr11 | 7172881 | 7173320 | 2.643237 | 4.23E-07 | down | + | -2.17246058134 | 0.00005 | down |
