# Supplementary figures and images for: N1-Methyladenosine modification of mRNA regulates neuronal gene expression and oxygen glucose deprivation/reoxygenation induction
Source: Cell Death Discov. 2023 May 12;9:159. doi: 10.1038/s41420-023-01458-2 (PMC10182019; doi:10.1038/s41420-023-01458-2)

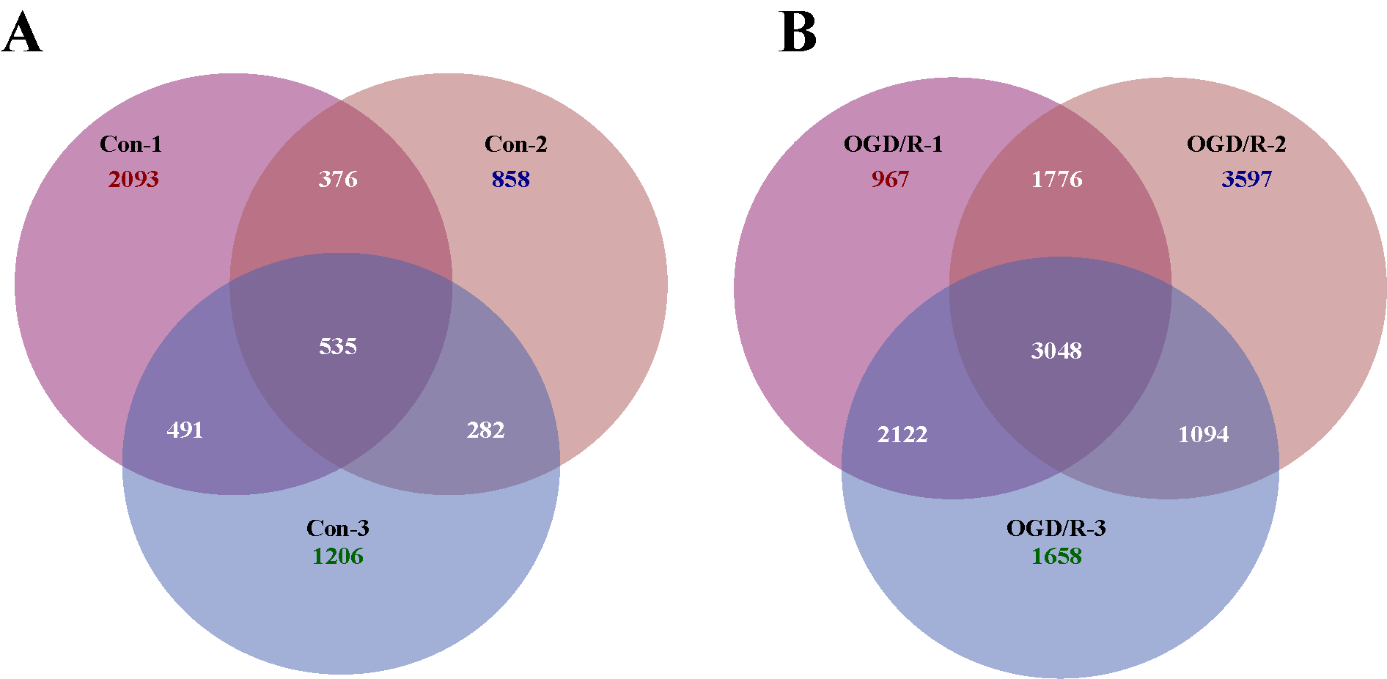

Supplement: Supplementary file 2 — Supplementary Figure 1 [file 41420_2023_1458_MOESM2_ESM.tif]

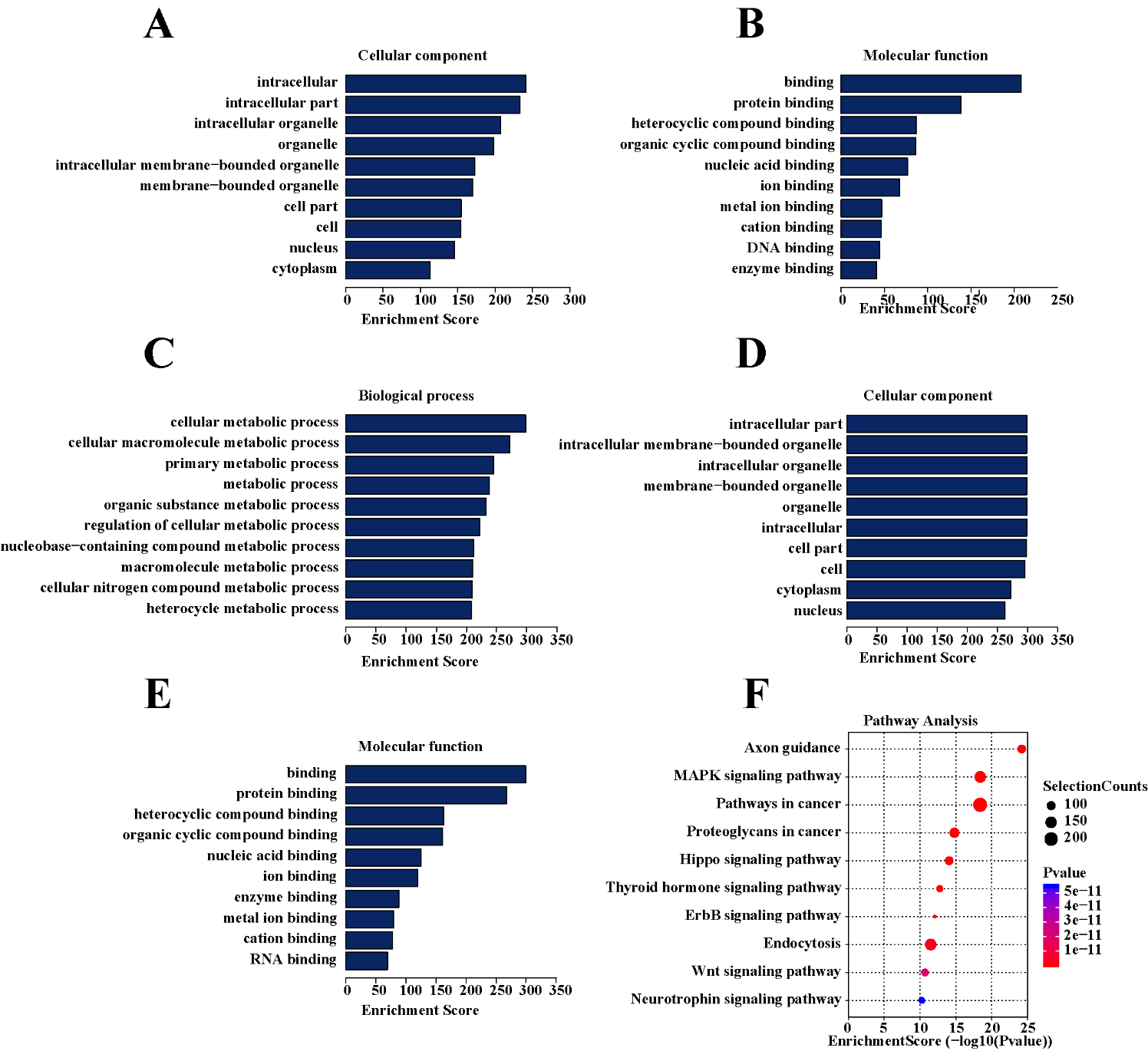

Supplement: Supplementary file 3 — Supplementary Figure 2 [file 41420_2023_1458_MOESM3_ESM.tif]

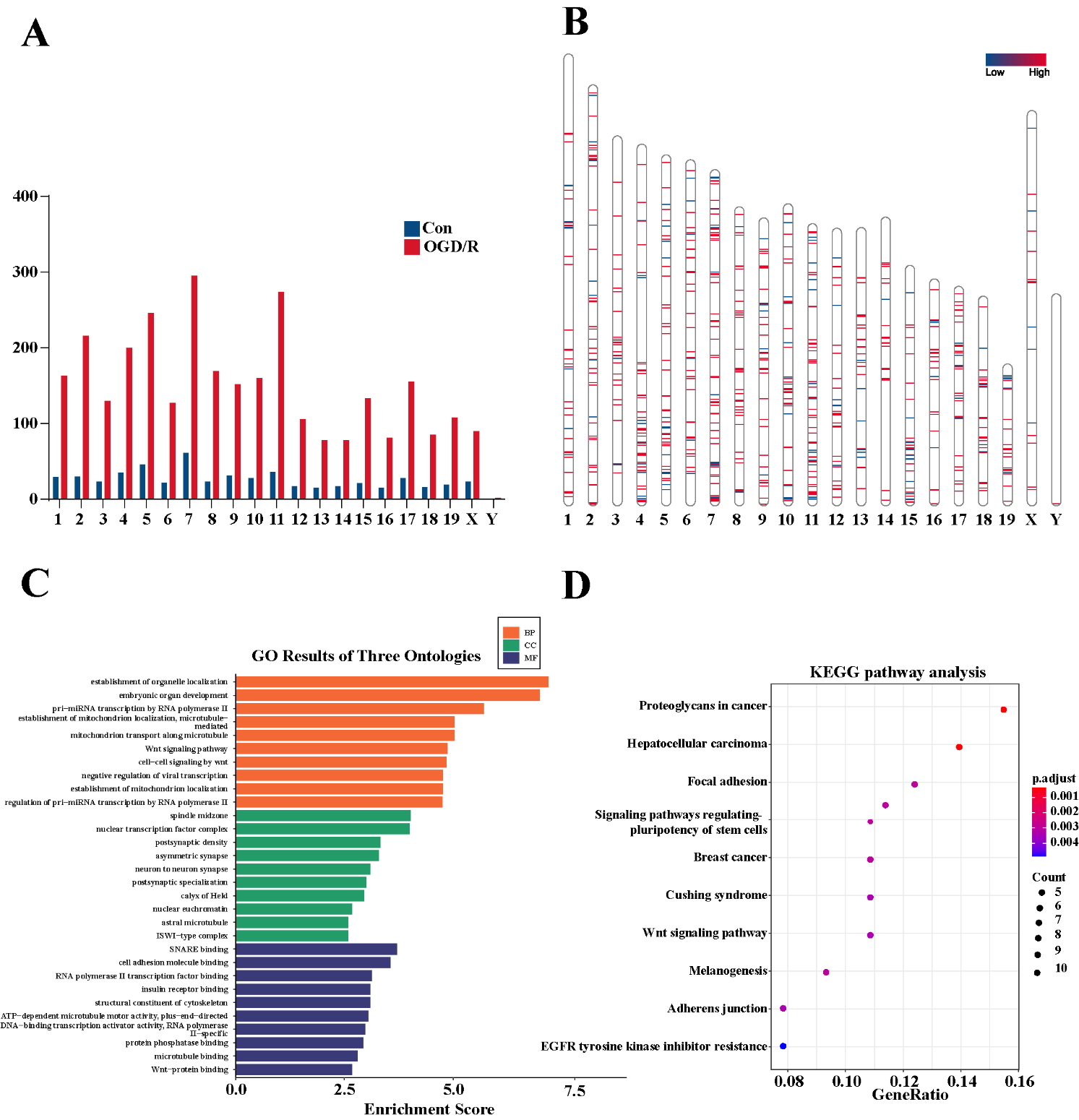

Supplement: Supplementary file 4 — Supplementary Figure 3 [file 41420_2023_1458_MOESM4_ESM.tif]

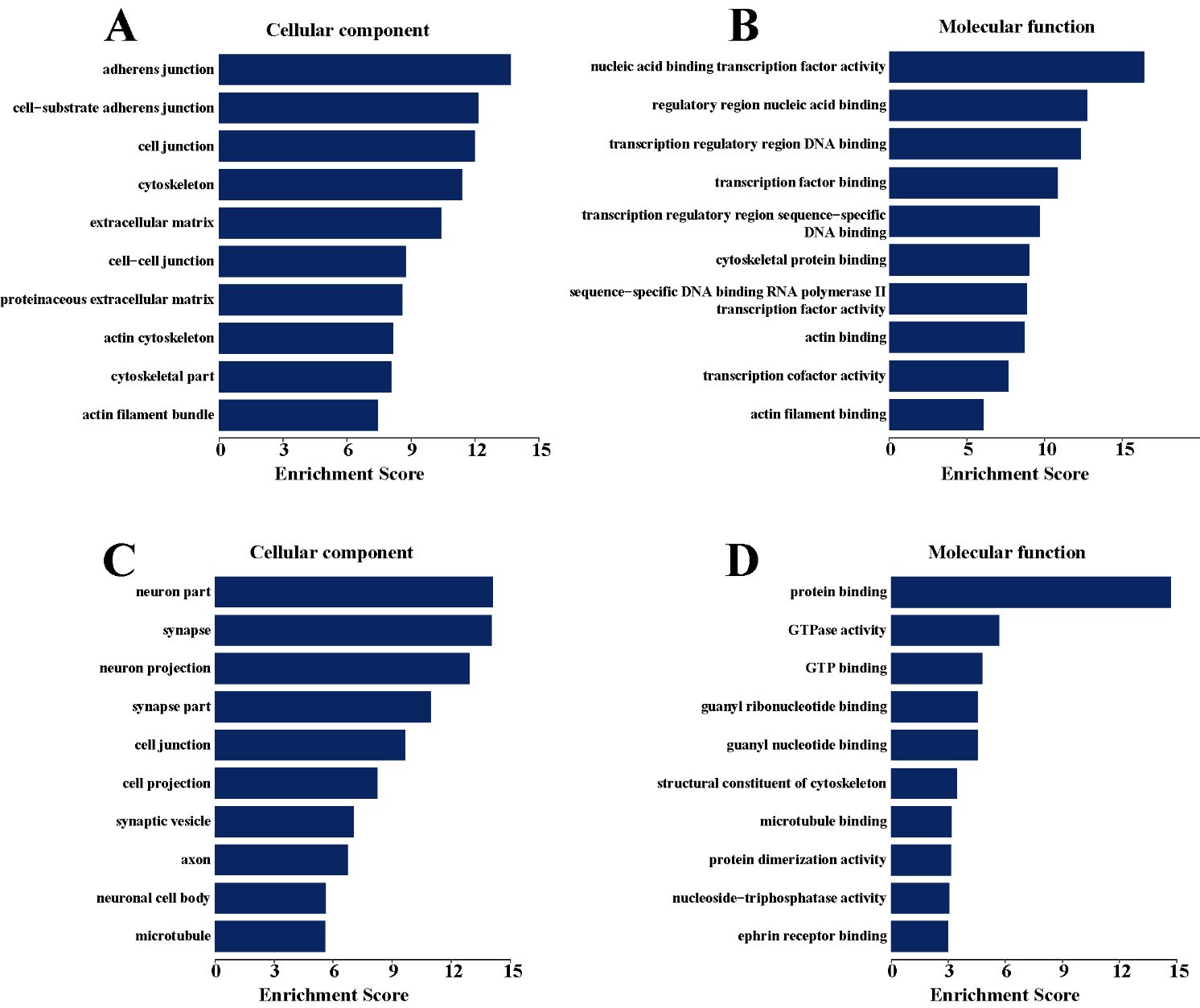

Supplement: Supplementary file 5 — Supplementary Figure 4 [file 41420_2023_1458_MOESM5_ESM.tif]

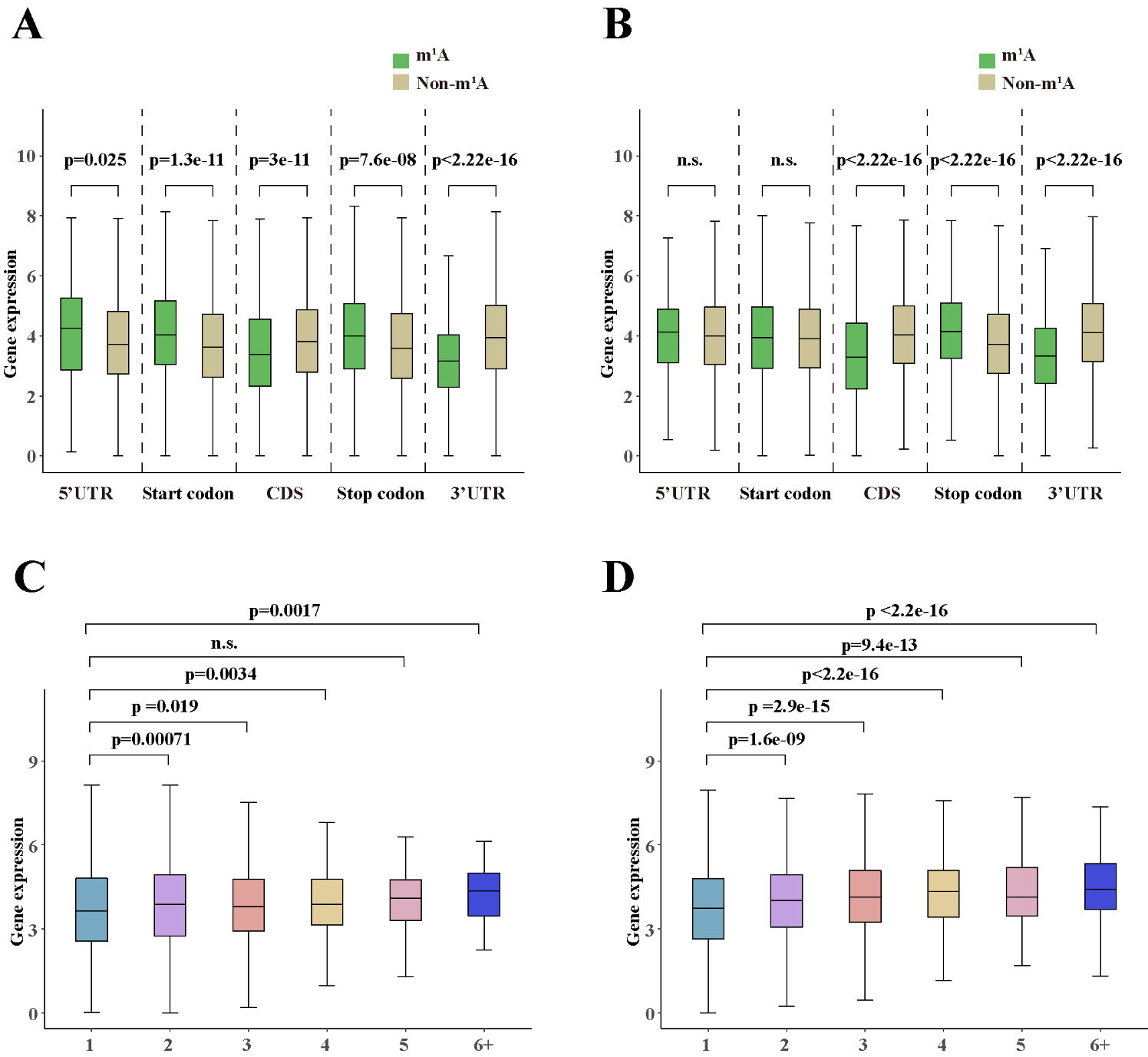

Supplement: Supplementary file 6 — Supplementary Figure 5 [file 41420_2023_1458_MOESM6_ESM.tif]

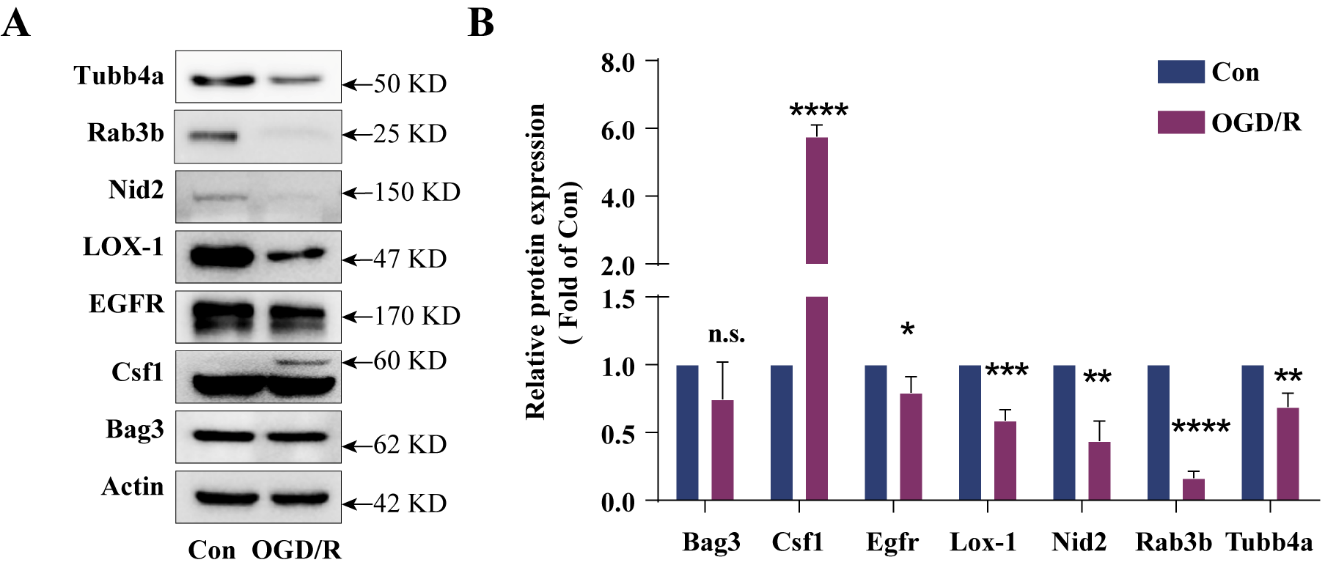

Supplement: Supplementary file 7 — Supplementary Figure 6 [file 41420_2023_1458_MOESM7_ESM.tif]

Figure 1 H

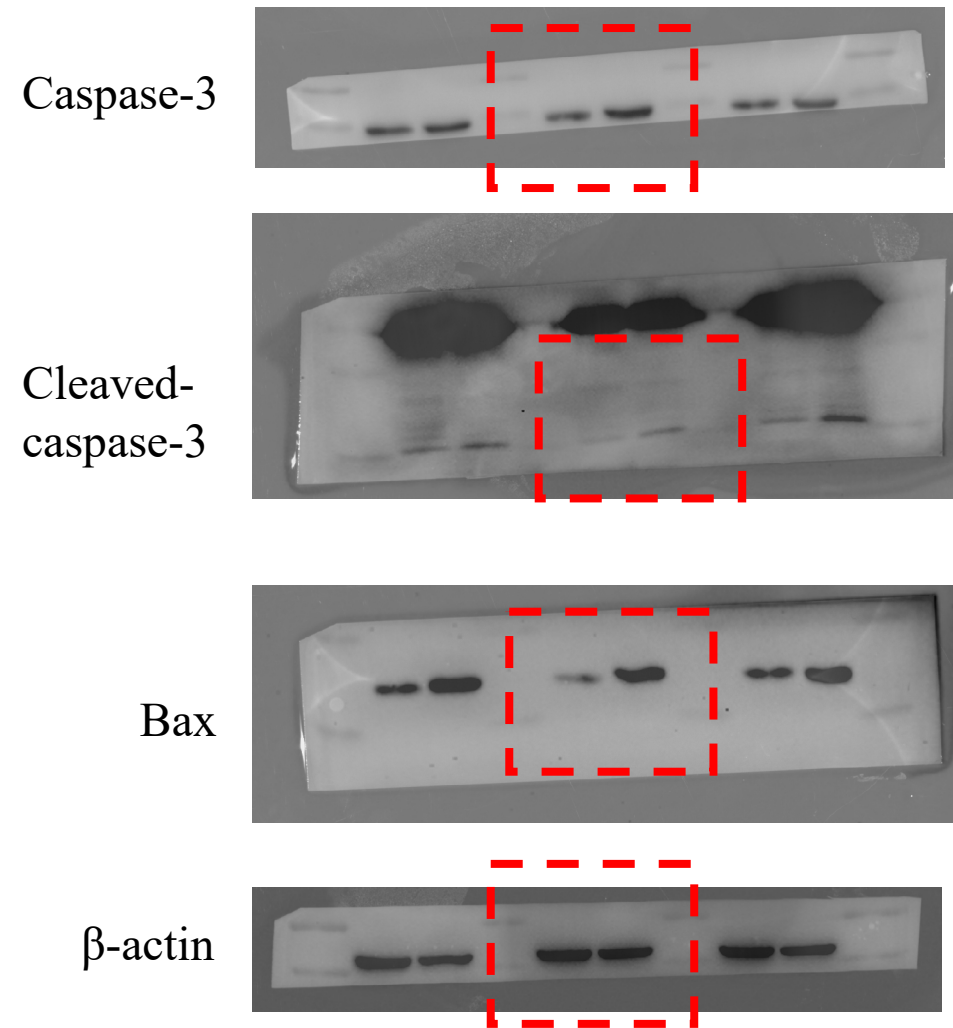

Figure 3I

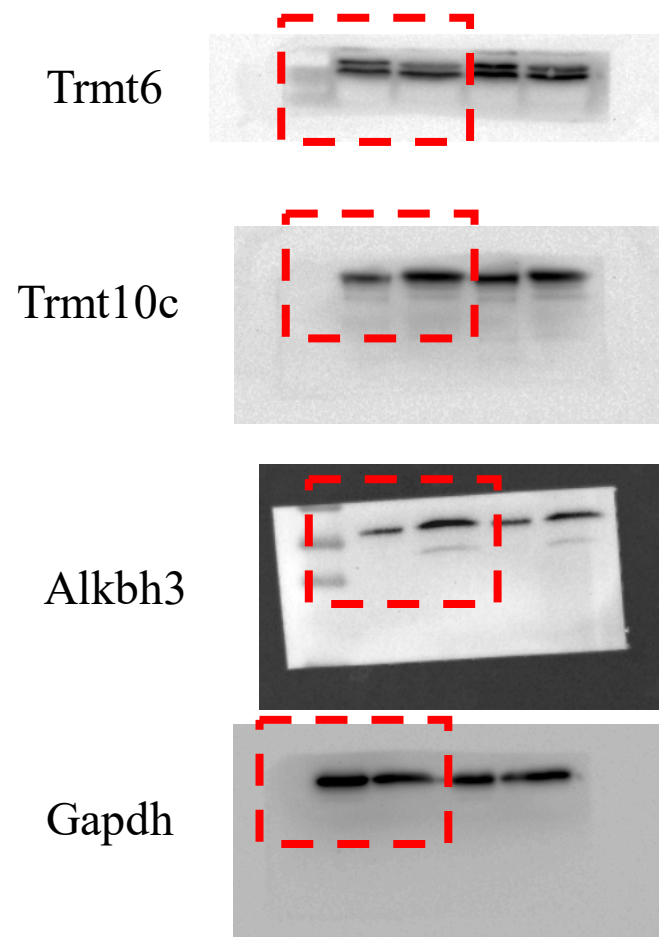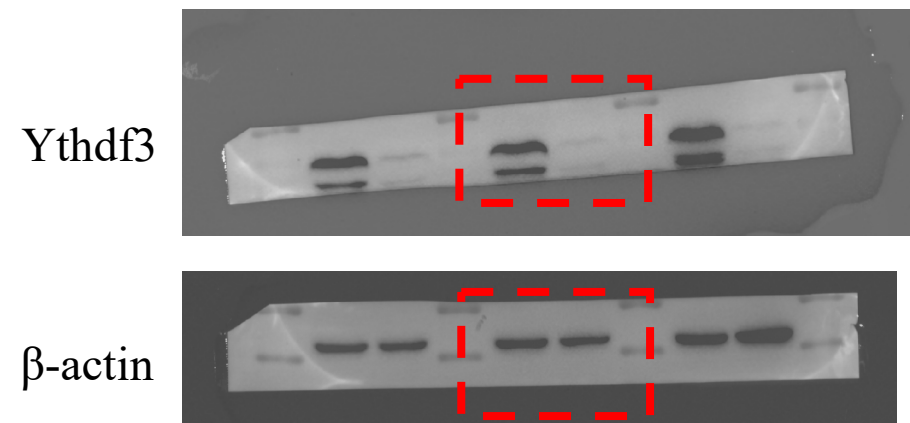

Supplementary Figure 6

Bag3

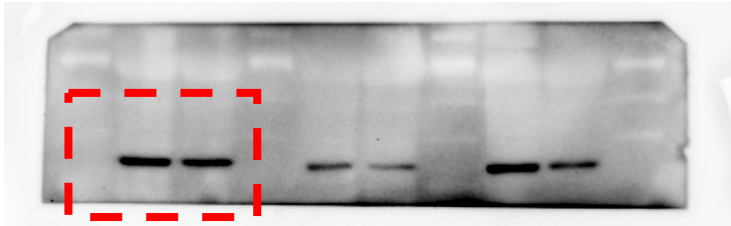

Egfr

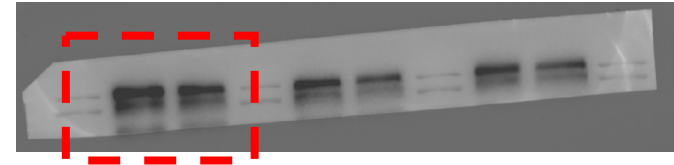

Csf1

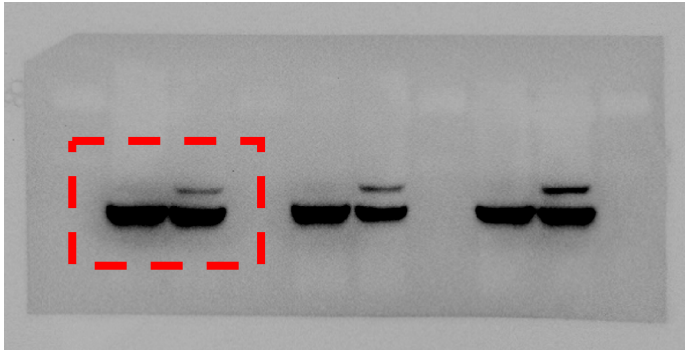

Rab3b

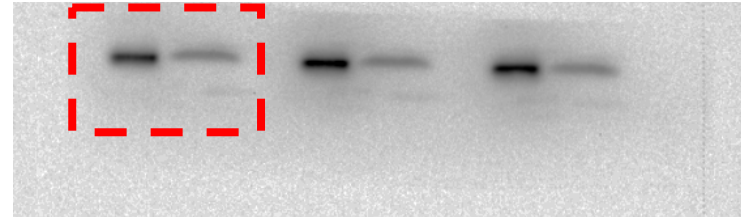

Lox-1

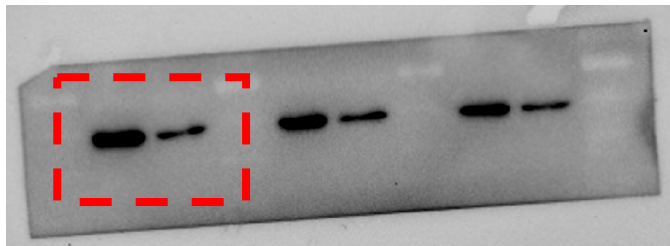

Tubb4a

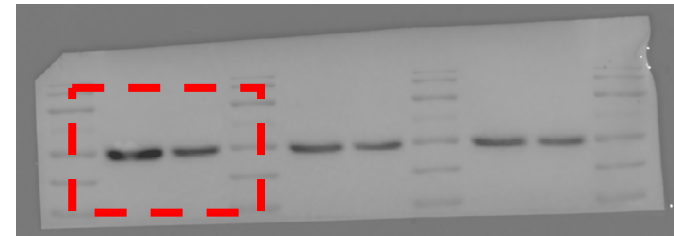

Nid2

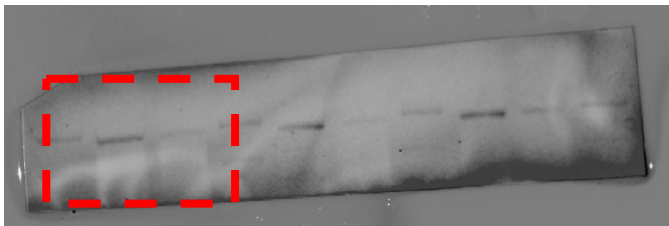

$\beta$ -actin

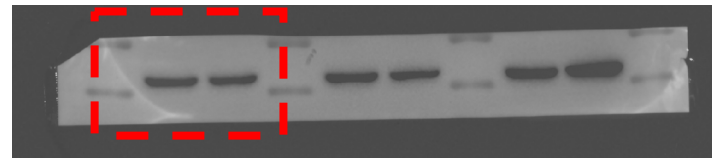

Supplement: Supplementary file 9 — Original Data File [file 41420_2023_1458_MOESM9_ESM.pdf]
