# Supplementary material for: N1-Methyladenosine modification of mRNA regulates neuronal gene expression and oxygen glucose deprivation/reoxygenation induction
Source: Cell Death Discov. 2023 May 12;9:159. doi: 10.1038/s41420-023-01458-2 (PMC10182019; doi:10.1038/s41420-023-01458-2)
Supplement: Supplementary file 8 — Supplementary Figure and Table Legends [file 41420_2023_1458_MOESM8_ESM.docx]

**Supplementary Figure 1.** Venn diagram of the number of methylation peaks in 3 biological replicates. (A): Control group; (B): OGD/R induction group.

**Supplementary Figure 2**. GO analysis (top 10) and KEGG analysis (top 10) of the m1A-modified genes in primary mouse cortical neurons and after OGD/R induction. (A), (B) GO analysis of the m1A-modified genes in primary mouse cortical neurons. (C), (D) GO analysis of the m1A-modified genes after OGD/R induction. (E) KEGG analysis of the m1A-modified genes during OGD/R induction. All data are presented as enrichment scores and p values.

**Supplementary Figure 3.** Distribution of m1A peaks on chromosomes. (A) Number distribution of m1A peaks on different chromosomes in mouse cortical neurons and OGD/R-induced transcripts. (B) Position distribution of differential m1A peaks on chromosomes after OGD/R induction. (C) GO analysis of the top 3 chromosomes with the greatest degrees of differential m1A gene expression levels. (D) KEGG analysis of the top 3 chromosomes with the greatest degrees of differential m1A gene expression levels. All data are presented as enrichment scores and p values.

**Supplementary Figure 4**. Differential m1A modification peaks are enriched in pathways related to neurological diseases and oxidative stress damage. (A), (B) GO analysis of genes with upregulated m1A modification peaks. (C), (D) GO analysis of genes with downregulated m1A modification peaks.

**Supplementary Figure 5.** Different positions and numbers of m1A peaks in the transcript have different effects on gene expression. (A), (B) m1A peaks at different positions in the transcript have different effects on gene expression. (C), (D) Changes in the numbers of m1A peaks in the transcript can regulate gene expression. A, C: Control group; B, D: OGD/R induction group. The box limits represent the upper quartile, the median and the lower quartile. The extremes represent the maximum and minimum values. ns: no significant difference. The p value was calculated using Student’s t-test.

**Supplementary Figure 6.** (A), (B) Western Blot shows changes in the expression of specific proteins during OGD/R induction. The data are presented as the means± SD, n = 3. The p value was calculated using Student’s t-test, *p<0.05, **p<0.01, ***p<0.0001. n.s.: no significant difference.

**Supplementary Table1.** Quality control information of the extracted RNA.

**Supplementary Table 2.** MeRIP Sequencing library quality control information.

**Supplementary Table 3.** Q30 library quality control information.

**Supplementary Table 4.** List of top 10 genes that exhibit a significant change upregulation in OGD/R induction in mouse primary cortical neurons.

**Supplementary Table 5.** List of top 10 genes that exhibit a significant change downregulation in OGD/R induction in mouse primary cortical neurons.
